# Periodontitis, Endothelial Dysfunction, and Systemic Inflammation: A Systematic Review and Meta-Analysis of Flow-Mediated Dilation

**DOI:** 10.3390/biomedicines14051106

**Published:** 2026-05-14

**Authors:** Cristina Ples, Cristina Savencu, Ana-Maria Pah, Gheorghe Stoichescu-Hogea, Diana-Maria Mateescu, Roxana Oancea

**Affiliations:** 1 Doctoral School, Department of Dental Medicine, “Victor Babes” University of Medicine and Pharmacy, Eftimie Murgu Square 2, 300041 Timisoara, Romania; cristina.stoichescu-hogea@umft.ro; 2 Department of Dental Prostheses Technology, Faculty of Dentistry, “Victor Babes” University of Medicine and Pharmacy, Eftimie Murgu Square 2, 300041 Timisoara, Romania; cristina.savencu@umft.ro; 3 Cardiology Department, “Victor Babes” University of Medicine and Pharmacy, Eftimie Murgu Square 2, 300041 Timisoara, Romania; 4 Doctoral School, Department of General Medicine, “Victor Babes” University of Medicine and Pharmacy, Eftimie Murgu Square 2, 300041 Timisoara, Romania; diana.mateescu@umft.ro; 5 Department of Preventive and Community Dentistry, “Victor Babes” University of Medicine and Pharmacy, Eftimie Murgu Square 2, 300041 Timisoara, Romania; roancea@umft.ro

**Keywords:** periodontitis, periodontal therapy, endothelial dysfunction, flow-mediated dilation, cardiovascular risk, systemic inflammation, C-reactive protein, interleukin-6, vascular function, meta-analysis

## Abstract

**Background/Objectives**: Periodontal disease is a chronic inflammatory condition associated with systemic vascular dysfunction and elevated cardiovascular risk. This systematic review and meta-analysis aimed to quantitatively evaluate the association between periodontitis and endothelial dysfunction and to assess the effects of periodontal therapy on endothelial function and inflammatory biomarkers. **Methods**: Conducted per PRISMA 2020 and registered in PROSPERO (CRD420261309247). Electronic databases (PubMed, Scopus, Cochrane Library) were searched for observational and interventional studies assessing endothelial function in patients with periodontitis. Risk of bias was evaluated using RoB 2, ROBINS-I, and the Newcastle–Ottawa Scale; certainty of evidence was assessed with GRADE. Pooled effects on flow-mediated dilation (FMD) and inflammatory markers were estimated using random-effects meta-analysis (DerSimonian–Laird). **Results**: Fourteen studies were included in qualitative synthesis; six contributed quantitative FMD data. Observational studies consistently demonstrated impaired endothelial function and elevated inflammatory markers in patients with periodontitis versus controls. Meta-analysis showed that periodontal therapy significantly improved endothelial function (pooled FMD: +3.3 percentage points; 95% CI 1.7–4.9; I^2^ = 77%; *p* < 0.001), though results should be interpreted cautiously given substantial heterogeneity and the limited number of studies (*n* = 6). Periodontal treatment reduced CRP levels (mean difference −0.38 mg/L; I^2^ = 41%); IL-6 showed a favorable direction but with very low certainty of evidence. Publication bias could not be reliably assessed given the small number of included studies. **Conclusions**: Periodontitis is associated with impaired endothelial function and systemic inflammation. Periodontal therapy leads to measurable improvements in both, supporting its role as a potentially modifiable contributor to cardiovascular risk. Larger randomized trials with long-term cardiovascular endpoints are warranted.

## 1. Introduction

Endothelial dysfunction occurs early in the atherothrombotic process and represents a central mechanistic link between cardiovascular risk factors and clinical events [1,2,3]. Periodontitis, a chronic low-grade inflammatory disease driven by dysbiotic subgingival biofilm and sustained host immune activation, leads to recurrent bacteremia, endotoxemia, and systemic dissemination of proinflammatory mediators such as C-reactive protein (CRP), interleukin-6 (IL-6), and tumor necrosis factor-α [4,5,6]. These mediators promote endothelial activation, oxidative stress, reduced nitric oxide bioavailability, and a pro-atherogenic vascular phenotype [5,7].

Consequently, individuals with periodontitis consistently show impaired endothelial function—most commonly measured by brachial artery flow-mediated dilation (FMD)—compared with periodontally healthy controls. Periodontal therapy has been associated with FMD improvement and reductions in circulating inflammatory biomarkers (CRP, IL-6, fibrinogen) [7,8,9,10,11,12]. However, the magnitude, consistency, and durability of these effects remain uncertain due to heterogeneity in study design, periodontal case definitions, treatment intensity, and timing of vascular assessments.

Previous systematic reviews have often lacked PRISMA 2020 compliance, structured risk-of-bias assessment, or GRADE evaluation, and frequently analyzed endothelial function and inflammatory markers separately [13,14,15]. To address these limitations, the present systematic review and meta-analysis provides an updated, methodologically rigorous quantitative synthesis of the association between periodontitis and endothelial dysfunction, focusing specifically on FMD as the primary functional outcome, together with key inflammatory biomarkers.

## 2. Materials and Methods

### 2.1. Study Design and Reporting Standards

This systematic review and meta-analysis was conducted in accordance with the Preferred Reporting Items for Systematic Reviews and Meta-Analyses (PRISMA 2020) guidelines and followed established methodological recommendations for quantitative evidence synthesis [16]. A completed PRISMA checklist is provided in Supplementary Table S1.

The study protocol was prospectively registered in the International Prospective Register of Systematic Reviews (PROSPERO) under registration number: CRD420261309247, prior to completion of the review, in line with recommendations for transparency and methodological rigor in systematic reviews [17]. No major deviations from the registered protocol occurred during the conduct of the study.

To ensure methodological transparency and reproducibility, the review followed a predefined protocol registered in PROSPERO and adhered to PRISMA 2020 reporting standards. The methodological workflow included systematic literature search, duplicate removal, independent screening of titles and abstracts, full-text eligibility assessment, standardized data extraction, risk-of-bias evaluation using validated tools according to study design, and quantitative synthesis using random-effects meta-analysis. All stages of the review process were performed independently by two investigators, with discrepancies resolved through discussion and consensus.

### 2.2. Research Questions and PICO Framework

The review addressed two primary research questions structured according to the Population–Intervention–Comparator–Outcome (PICO) framework, a widely accepted approach for defining eligibility criteria and guiding systematic reviews [18].

The components of the PICO framework were defined as follows: Population (P): adults with clinically diagnosed periodontitis or periodontal disease. Intervention (I): periodontal therapy, including non-surgical periodontal treatment such as scaling and root planing or intensive periodontal treatment protocols. Comparator (C): periodontally healthy individuals or control groups receiving standard care, less intensive periodontal therapy, or baseline measurements in within-subject interventional studies. Outcome (O): endothelial function assessed using validated vascular techniques, primarily flow-mediated dilation (FMD) of the brachial artery or forearm blood flow plethysmography, together with systemic inflammatory biomarkers such as C-reactive protein (CRP) and interleukin-6 (IL-6).

Periodontal diagnoses reported in the included studies were based on the classification systems available at the time of the original investigations, most commonly the 1999 American Academy of Periodontology (AAP) classification. Across the included studies, periodontitis was generally defined using combinations of clinical attachment loss (CAL), probing pocket depth (PPD), bleeding on probing (BoP), and radiographic evidence of alveolar bone loss. Although the diagnostic thresholds varied slightly between studies, they generally corresponded to moderate-to-severe periodontal disease according to contemporary definitions. Because most studies were conducted prior to the introduction of the 2017 AAP/European Federation of Periodontology (EFP) classification system, direct staging and grading were not reported. However, based on the severity of attachment loss and pocket depth described in the original publications, the periodontal conditions included in this review broadly correspond to Stage II–III periodontitis under the 2017 classification framework.

The primary outcome of the review was endothelial function assessed by validated functional vascular measurements, particularly flow-mediated dilation (FMD). Structural vascular markers such as carotid intima–media thickness (CIMT) were not predefined as primary or secondary outcomes for quantitative synthesis in the protocol and were therefore reported descriptively when available in the included studies. Secondary outcomes included systemic inflammatory biomarkers associated with periodontal inflammation and vascular dysfunction, including C-reactive protein (CRP) and interleukin-6 (IL-6).

### 2.3. Eligibility Criteria

Eligibility criteria were defined a priori based on methodological recommendations for systematic reviews and meta-analyses of clinical studies [18]. Observational studies, including cross-sectional and case–control designs, were considered eligible when they evaluated endothelial function using validated vascular techniques and included an appropriate comparator group. Cross-sectional studies were not excluded a priori; however, studies lacking direct endothelial function measurements or suitable comparator groups were excluded during full-text assessment.

### 2.4. Information Sources and Search Strategy

The search strategy was developed following established guidance for systematic literature searches in biomedical databases and systematic reviews [19]. The detailed search strategy for each database is provided in Supplementary Table S2. To minimize the risk of missing relevant studies, search strategies were adapted to the specific indexing structure of each database. For PubMed and Scopus, a comprehensive combination of controlled vocabulary terms (MeSH/Emtree) and free-text keywords was used. For the Cochrane Library, the search strategy incorporated broader keyword combinations reflecting the database’s indexing system for clinical trials and systematic reviews. The search terms were iteratively refined and combined using Boolean operators to maximize sensitivity. Google Scholar was not used as a primary database for systematic searching because its search algorithms are dynamic and may limit reproducibility. Instead, it was used only for supplementary citation tracking and exploratory screening of potentially relevant studies. The detailed search strategies for each database are provided in Supplementary Table S2.

To reduce the risk of missing relevant evidence, supplementary screening of reference lists of eligible articles and relevant reviews was performed. Grey literature sources such as conference abstracts and non–peer-reviewed reports were not systematically included because these sources rarely provide sufficient methodological detail or extractable numerical data required for quantitative meta-analysis of endothelial function outcomes.

### 2.5. Study Screening and Selection Process

The study selection process was conducted in accordance with PRISMA recommendations [16].

### 2.6. Data Extraction

Data extraction was performed using a standardized form based on methodological recommendations for systematic reviews of clinical studies [18].

Data extraction was performed independently by two reviewers using a standardized extraction form developed for the present review. Extracted variables included study design, population characteristics, periodontal diagnostic criteria, intervention details, endothelial function assessment methods, inflammatory biomarkers, follow-up duration, and numerical outcome data required for meta-analysis. Any discrepancies between reviewers were resolved by consensus.

### 2.7. Risk of Bias Assessment

Risk of bias was assessed independently by two reviewers using validated tools appropriate for study design: Randomized controlled trials: Cochrane Risk of Bias 2 (RoB 2) tool (Cochrane Handbook version 6.5; https://www.cochrane.org/, accessed on 1 April 2026) [20]; Non-randomized interventional studies: ROBINS-I tool (https://www.riskofbias.info/, accessed on 1 April 2026) [21]; Observational studies: Newcastle–Ottawa Scale (NOS) (available at: https://www.ohri.ca/programs/clinical_epidemiology/oxford.asp, accessed on 1 April 2026) [22].

Studies were categorized as low, moderate, or high risk of bias according to established thresholds.

The detailed risk of bias assessment is presented in Supplementary Table S3.

### 2.8. Data Synthesis and Statistical Analysis

Quantitative synthesis was performed using a random-effects meta-analysis model (DerSimonian–Laird method), which accounts for between-study heterogeneity [23]. A random-effects model was selected a priori because clinical and methodological heterogeneity across studies was anticipated, including differences in periodontal disease severity, treatment protocols, cardiovascular risk profiles, and follow-up duration. The DerSimonian–Laird estimator was used to account for between-study variance. Given methodological differences, within-group and between-group effect estimates were not pooled directly but interpreted within a unified random-effects framework with sensitivity analyses restricted to randomized comparisons. For non-randomized interventional studies, effect sizes were derived from within-group changes from baseline, whereas for randomized controlled trials, between-group differences at follow-up were used. These designs were synthesized within a single random-effects model, and sensitivity analyses restricted to randomized comparisons were performed to mitigate potential bias arising from within-group estimates. This mixed pooling approach was adopted because the available evidence in this field consists predominantly of non-randomized interventional studies; restricting the analysis exclusively to randomized controlled trials would have reduced the quantitative synthesis to only two trials (Tonetti et al. [24] and Okada et al. [25]), further limiting statistical precision. It must be acknowledged, however, that combining within-group pre–post changes with between-group RCT differences in a single pooled estimate may overestimate the true treatment effect, as within-group changes are susceptible to regression to the mean, placebo effects, and time-related confounding that are controlled for in randomized parallel-group designs. Readers should therefore weight the sensitivity analysis restricted to RCTs—which yielded a smaller, non-significant effect—as the more conservative and methodologically robust estimate.

Heterogeneity was assessed using the Cochran Q test and the I^2^ statistic, with thresholds interpreted according to conventional recommendations [26].

Publication bias was evaluated using funnel plots, Egger regression test, and Begg test, when sufficient studies were available [27].

Statistical analyses were performed using R software (version 4.3.2; R Foundation for Statistical Computing, Vienna, Austria) with the meta and metafor packages for calculation of pooled effect sizes, heterogeneity statistics, and sensitivity analyses. Review Manager (RevMan version 5.4; Cochrane Collaboration, Copenhagen, Denmark) was used primarily for graphical visualization of the meta-analysis results, including the generation of forest plots. All pooled estimates reported in the manuscript were derived from analyses performed in R, while RevMan was used solely for visualization purposes.

For endothelial function (FMD), only studies reporting numeric values suitable for effect size calculation were included in quantitative pooling, while studies without extractable numerical data were summarized narratively. When summary statistics required for meta-analysis (e.g., standard deviations or exact numerical values) were not reported in the original publications, attempts were made to derive these values from available information such as confidence intervals, *p*-values, or graphical data, when possible, following the methodological recommendations of the Cochrane Handbook for Systematic Reviews of Interventions.

When numerical data necessary for effect size calculation could not be reliably obtained, the corresponding studies were included in the qualitative synthesis but were not eligible for quantitative pooling. Because most of the studies were published more than a decade ago, attempts to contact study authors for missing data were considered unlikely to yield additional information and were therefore not systematically pursued. For studies reporting randomized or controlled comparisons, effect sizes were calculated using between-group differences in FMD.

For studies without a parallel control group, effect sizes were derived from within-group changes from baseline. Because these designs differ methodologically, the results were interpreted with caution and sensitivity analyses restricted to randomized controlled trials were performed to evaluate the robustness of the findings. To further evaluate the potential influence of study design on pooled estimates, a sensitivity analysis restricted to randomized controlled trials was performed. The direction of effect observed in the randomized studies was consistent with the overall pooled estimate, supporting the robustness of the findings despite the inclusion of non-randomized interventional studies. Given the heterogeneity of study designs, sensitivity analyses were performed to explore the influence of individual studies on pooled estimates and to assess the robustness of results when analyses were restricted to randomized controlled trials.

Inflammatory biomarkers were reported using the units provided in the original studies. Because IL-6 values were reported using heterogeneous units across studies (ng/L or pg/mL), results were standardized and reported uniformly in ng/L.

### 2.9. Certainty of Evidence (GRADE)

The certainty of evidence for each major outcome was evaluated using the Grading of Recommendations Assessment, Development and Evaluation (GRADE) framework [28,29].

Evidence quality was classified as high, moderate, low, or very low based on risk of bias, inconsistency, indirectness, imprecision, and publication bias.

GRADE summary tables are presented in Supplementary Table S4.

### 2.10. Ethical Considerations

Ethical approval was not required because this study analyzed previously published data without involving individual patient information.

## 3. Results

### 3.1. Study Selection

**Figure 1:**
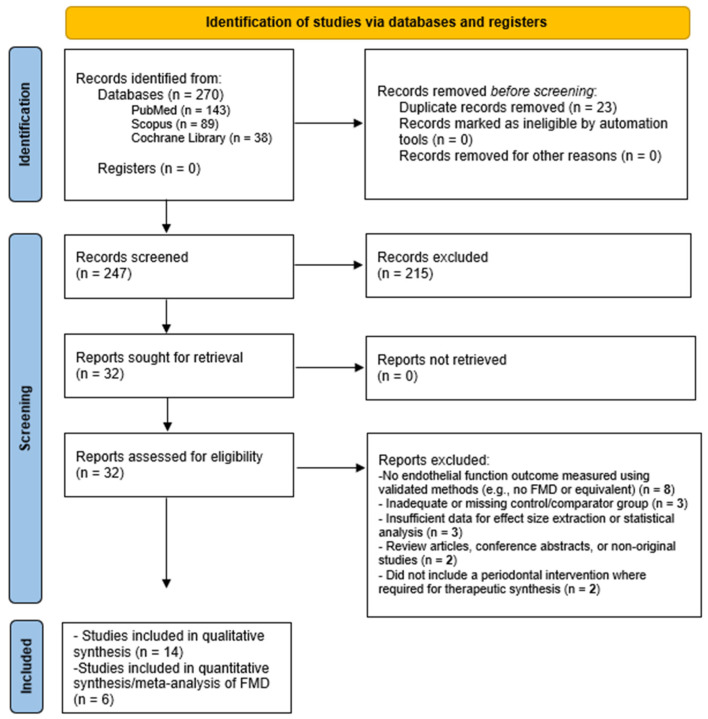
PRISMA 2020 flow diagram of the study selection process [24,25,30,31,32,33,34,35,36,37,38,39,40,41].

The systematic search retrieved 270 records (PubMed: *n* = 143, Scopus: *n* = 89, Cochrane Library: *n* = 38). After removal of 23 duplicates, 247 unique records were screened by title and abstract. Of these, 32 full-text articles were assessed for eligibility. Fourteen studies [24,25,30,31,32,33,34,35,36,37,38,39,40,41] met the predefined inclusion criteria and were included in the qualitative synthesis (Figure 1). Among these, studies reporting extractable quantitative data on endothelial function assessed by validated vascular techniques (e.g., flow-mediated dilation or plethysmography) were included in the quantitative meta-analysis. Studies reporting only systemic inflammatory biomarkers without direct endothelial function assessment were retained for qualitative synthesis but were not eligible for quantitative pooling. Several studies identified during full-text screening were excluded because they did not assess endothelial function using validated functional vascular measurements, did not include a periodontal intervention or appropriate comparator group, or did not report extractable quantitative data suitable for meta-analysis.

### 3.2. Study and Patient Characteristics

Fourteen studies [24,25,30,31,32,33,34,35,36,37,38,39,40,41] were included in the qualitative synthesis, comprising both observational and interventional designs. Among these, studies reporting extractable quantitative data on endothelial function assessed by validated vascular techniques were included in the meta-analysis.

The included studies were published between 2003 and 2025 and enrolled participants across diverse populations. Individual study sample sizes ranged from 13 to over 1000 participants, including 79 participants in Amar et al. [30], 40 in Mercanoglu et al. [32], 30 in Seinost et al. [33], 94 in D’Aiuto et al. [37], 120 in Tonetti et al. [24], 13 in Blum et al. [34], 96 in Higashi et al. [31], 40 in Piconi et al. [36], 77 in Vidal et al. [38], 110 in Okada et al. [25], 35 in Molina et al. [39], and over 1000 participants in Holtfreter et al. [41].

The diagnostic definitions of periodontitis varied slightly across studies, reflecting the classification systems available at the time of study conduct.

Mean age across studies ranged from approximately 35 to 58 years, and sex distribution was generally balanced. Populations included otherwise healthy adults with chronic periodontitis [24,30,32,33], hypertensive patients with and without periodontitis [31,38], cohorts with severe generalized periodontitis undergoing therapy [24,33,37], and mixed cardiovascular-risk populations [34,36,39].

Endothelial function was assessed using validated vascular techniques, most commonly brachial artery flow-mediated dilation, while some studies employed alternative physiological methods such as forearm blood flow plethysmography to evaluate endothelium-dependent vasodilation.

It should be noted that several studies included in this review were conducted prior to the 2017 AAP/EFP World Workshop classification of periodontal diseases. Consequently, periodontal diagnoses were reported using earlier terminology (e.g., chronic or severe periodontitis). In the context of the current classification framework, these categories would generally correspond to Stage II–IV periodontitis depending on disease severity and extent. Because individual studies did not provide sufficient clinical detail to allow precise retrospective staging and grading, the original diagnostic terminology reported by the authors was retained to preserve methodological consistency.

Study and patient characteristics are summarized in Table 1.

### 3.3. Assessment of Endothelial Function

Endothelial function was evaluated using validated vascular physiology techniques across the included studies. The most commonly used method was brachial artery flow-mediated dilation (FMD), a well-established non-invasive marker of endothelial nitric oxide bioavailability [24,25,30,32,33,34,36,39,40,41].

In addition to FMD-based assessments, Higashi et al. employed forearm blood flow plethysmography to quantify acetylcholine-stimulated (endothelium-dependent) and sodium nitroprusside-stimulated (endothelium-independent) vasodilation, thereby allowing differentiation between endothelial dysfunction and generalized vascular smooth muscle impairment [31]. In that study, endothelium-independent responses remained preserved, supporting the endothelial specificity of the observed abnormalities [31].

Some studies evaluated systemic inflammatory biomarkers without direct functional assessment of endothelial vasodilation [35,37,38]. These findings were considered in the qualitative synthesis but were not eligible for quantitative pooling of endothelial function outcomes.

Overall, despite methodological heterogeneity in vascular assessment techniques, the included studies consistently focused on validated measures of endothelial function, supporting the overall consistency of the observed association between periodontal disease and vascular dysfunction.

### 3.4. Association Between Periodontitis and Endothelial Dysfunction

Observational data consistently demonstrated that periodontitis is associated with impaired endothelial function and increased systemic inflammation, although the strength of the association varied according to disease severity and population characteristics.

In the case–control study by Amar et al. including 79 participants, FMD was significantly lower in patients with periodontitis compared with healthy controls (7.8 ± 4.6% vs. 11.7 ± 5.3%; *p* = 0.005), corresponding to an approximate 33% relative reduction in endothelial function. C-reactive protein (CRP) levels were also significantly higher in participants with periodontitis (*p* < 0.01) [30].

In the mechanistic study by Higashi et al. (96 participants), periodontitis was associated with significantly blunted acetylcholine-induced forearm vasodilation in both normotensive and hypertensive individuals (*p* < 0.01), whereas sodium nitroprusside responses remained unchanged [31]. Periodontitis groups also exhibited higher circulating CRP and interleukin-6 (IL-6) concentrations (both *p* < 0.01), supporting an inflammation-mediated mechanism of endothelial dysfunction that appears additive to the effects of hypertension [31].

Similar findings were observed in larger population-based and case–control studies. Holtfreter et al., in a cross-sectional analysis of 1234 participants from the general population, demonstrated that periodontitis was associated with impaired brachial artery FMD, independent of traditional cardiovascular risk factors [41]. Velosa-Porras et al. found lower FMD values in adults with stage III–IV periodontitis compared with periodontally healthy or gingivitis controls, although the difference did not always reach statistical significance after full adjustment [40]. These observational results support the presence of endothelial impairment linked to periodontal inflammation across different settings and severity levels.

### 3.5. Effects of Periodontal Therapy on Endothelial Function

Interventional studies demonstrated that periodontal therapy may be associated with improvements in endothelial function, although the magnitude and temporal patterns of the effect varied considerably according to baseline disease severity, treatment intensity, follow-up duration, and cardiovascular risk profile of the participants.

In the randomized controlled trial by Tonetti et al. including 120 patients with severe generalized periodontitis, intensive periodontal therapy induced a biphasic vascular response. At 24 h, FMD showed a transient reduction in the intensive-treatment group (absolute between-group difference −1.4%; 95% CI −2.3 to −0.5; *p* = 0.002), consistent with acute inflammatory activation. By 60 days, FMD improved (between-group difference +0.9%; 95% CI 0.1–1.7; *p* = 0.02), and by 180 days the between-group difference reached +2.0% (95% CI 1.2–2.8; *p* < 0.001), indicating sustained endothelial recovery [24].

Mercanoglu et al. evaluated 40 patients with chronic periodontitis and reported a marked increase in FMD after initial non-surgical periodontal therapy (from 8.4 ± 4.0% to 17.7 ± 5.7%; absolute improvement +9.3%; *p* < 0.0001), representing more than a 100% relative gain in endothelial function in patients with severely impaired baseline values [32]. In the study by Seinost et al. of 30 patients with severe periodontitis, FMD increased from 6.1 ± 4.4% to 9.8 ± 5.7% (absolute change +3.7%; *p* = 0.003) after treatment, accompanied by a modest reduction in CRP [31]. Blum et al. also demonstrated significant post-treatment improvements in endothelial function (*p* < 0.05), supporting the reproducibility of vascular benefits across independent cohorts [34].

Piconi et al. extended these observations by assessing both functional and structural vascular indices over 12 months. Periodontal therapy was associated with significant improvement in FMD (*p* < 0.01) and a reduction in carotid intima–media thickness (*p* < 0.05), suggesting that sustained periodontal control may favorably influence early atherosclerotic remodeling in addition to endothelial function [36].

**Figure 2:**
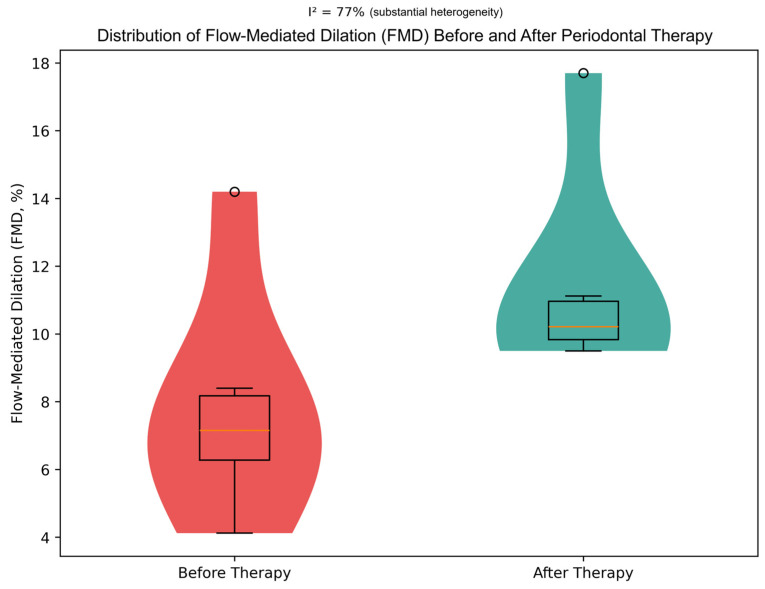
Distribution of flow-mediated dilation (FMD) values before and after periodontal therapy across included interventional studies [24,25,32,33,34,35,36,39]. Violin plots represent kernel density estimation of FMD values across included studies. Embedded boxplots indicate the median and interquartile range (IQR), with whiskers extending to 1.5 × IQR. Circles represent potential outlier values. The distribution illustrates increased FMD values following periodontal therapy, alongside substantial between-study heterogeneity (I^2^ = 77%).

In contrast, not all interventional studies demonstrated a significant effect on endothelial function. Okada et al., in an open-label randomized controlled trial of 110 patients with early-stage periodontal disease, found that advanced periodontal self-care for 3 months improved periodontal parameters but did not result in a statistically significant improvement in FMD compared with standard oral hygiene [25]. Similarly, in a randomized pilot trial by Molina et al. involving 35 patients with moderate-to-severe periodontitis and established cardiovascular disease, non-surgical periodontal therapy led to improved periodontal outcomes and a modest reduction in carotid intima–media thickness, but no significant intergroup difference was observed in FMD at follow-up [39]. To further illustrate the distribution and variability of endothelial function measurements across studies, a violin plot was constructed (Figure 2). This visualization highlights the marked heterogeneity of flow-mediated dilation (FMD) values before and after periodontal therapy, complementing the pooled estimates derived from the random-effects meta-analysis. The spread and density of values confirm substantial between-study variability, consistent with the observed heterogeneity (I^2^ = 77%).

Study-level changes in endothelial function are summarized in Table 2.

### 3.6. Effects of Periodontal Therapy on Systemic Inflammation

Interventional trials generally reported reductions in systemic inflammatory markers following successful periodontal therapy, although the magnitude and timing of these changes varied depending on baseline inflammatory burden, treatment intensity, and patient cardiovascular risk profile.

In a randomized trial of 94 patients with severe periodontitis, D’Aiuto et al. observed significant decreases at 6 months in IL-6 (mean reduction 0.2 ng/L; 95% CI 0.1–0.4; *p* = 0.001) and CRP (mean reduction 0.5 mg/L; 95% CI 0.4–0.7; *p* < 0.0001) [37]. Participants with the greatest periodontal clinical improvement experienced the largest declines in inflammatory biomarkers, suggesting a dose–response relationship between local periodontal control and systemic inflammation.

In patients with severe periodontitis and refractory hypertension, Vidal et al. demonstrated significant reductions in IL-6, CRP, and fibrinogen after periodontal therapy (all *p* < 0.05), indicating that anti-inflammatory effects may extend to high cardiovascular-risk populations [38]. Ide et al. reported an early, short-lived increase in IL-6 and tumor necrosis factor-α immediately after scaling and root planing, followed by a return toward baseline levels. This transient rise is consistent with an acute procedural inflammatory response that precedes longer-term systemic improvement [35].

In a randomized pilot trial involving 35 patients with periodontitis and established cardiovascular disease, Molina et al. reported favorable trends toward reduction in systemic inflammatory markers following non-surgical periodontal therapy, although between-group differences were not consistently statistically significant [39]. These findings are consistent with a potential systemic anti-inflammatory effect of periodontal treatment, even in patients with preexisting cardiovascular disease.

Study-level changes in systemic inflammatory markers are summarized in Table 3.

### 3.7. Quantitative Meta-Analysis

A random-effects meta-analysis using the DerSimonian–Laird method was performed to quantitatively synthesize the effect of periodontal therapy on the primary outcome, flow-mediated dilation (FMD) of the brachial artery. Six interventional studies provided sufficient extractable numerical data on FMD and were included in the quantitative pooling: Mercanoglu et al. (2004) [32], Seinost et al. (2005) [33], Tonetti et al. (2007) [24], Blum et al. (2007) [34], Piconi et al. (2009) [36], and Okada et al. (2021) [25]. Effect sizes were derived from within-group changes in non-randomized studies (Mercanoglu [32], Seinost [33], Blum [34], Piconi [36]) and from between-group differences in randomized controlled trials (Tonetti [24], Okada [25]). Sensitivity analyses restricted to randomized controlled trials (Tonetti et al. [24], Okada et al. [25], and Molina et al. [39]) showed a smaller and non-significant effect on FMD, indicating that the overall pooled estimate is largely influenced by earlier non-randomized studies [32,33,34,36].

The pooled mean difference in FMD following periodontal therapy was +3.3 percentage points (95% CI 1.7–4.9; *p* < 0.001; I^2^ = 77%). Moderate-to-high statistical heterogeneity (I^2^ = 77%) was observed, largely attributable to differences in baseline periodontal disease severity, treatment intensity (intensive versus conventional non-surgical therapy), follow-up duration, and the cardiovascular risk profile of the included populations. Notably, earlier trials enrolling patients with more severe periodontitis and markedly impaired baseline endothelial function generally reported larger improvements, whereas the more recent trial by Okada et al. (2021) [25] showed a smaller, non-significant effect, contributing to a more conservative overall pooled estimate.

Observational studies (Amar et al. 2003 [30], Higashi et al. 2008 [31], Holtfreter et al. 2013 [41], Velosa-Porras et al. 2021 [40]) and the randomized pilot trial by Molina et al. (2025) [39], which reported a neutral effect on FMD, were not included in the quantitative pooling. These studies were retained only in the qualitative (narrative) synthesis and are presented descriptively in Table 2. This approach provides a balanced overview of the available evidence, integrating both the substantial improvements observed in earlier interventional trials and the more conservative findings from contemporary studies.

**Figure 3:**
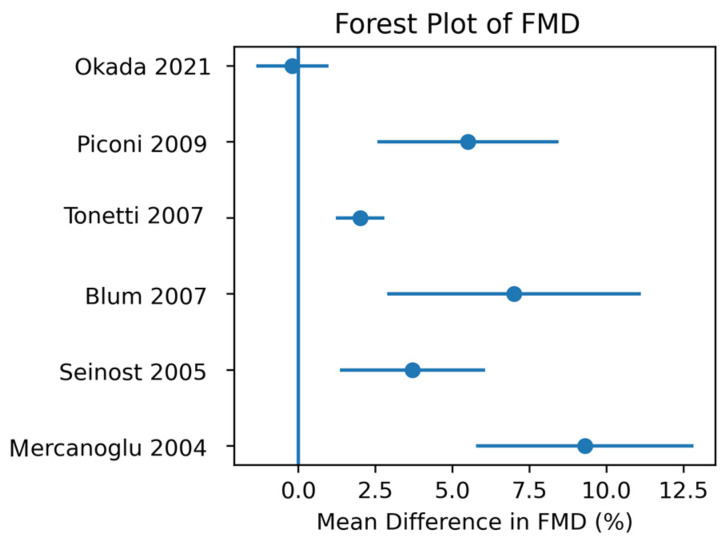
Forest plot showing the effects of periodontal therapy on endothelial function, expressed as mean differences in flow-mediated dilation (FMD) in percentage points [24,25,32,33,34,36]. Individual study estimates are represented by squares, with horizontal lines indicating 95% confidence intervals. The size of each square reflects the inverse-variance weight of the corresponding study. The diamond represents the pooled effect estimate calculated using a random-effects model (DerSimonian–Laird method). Positive values indicate improvement in endothelial function following periodontal therapy.

Sensitivity analyses restricted to randomized controlled trials (where data permitted) confirmed a consistent direction of effect, although the magnitude of the improvement in FMD was modestly attenuated compared with the overall pooled estimate. Leave-one-out analyses did not materially alter the pooled mean difference, supporting the robustness of the findings despite the presence of clinical and methodological heterogeneity. The individual study estimates and the overall pooled effect from the six interventional trials are presented in Figure 3.

Owing to the small number of studies (*n* = 6) providing extractable FMD data and the substantial statistical heterogeneity (I^2^ = 77%), the pooled effect size should be considered exploratory and indicative of the direction of effect rather than a precise estimate of the treatment effect. The pooled improvement in FMD appeared to be driven predominantly by earlier studies enrolling patients with severe periodontitis and markedly impaired baseline endothelial function, in which large absolute gains were observed. By contrast, more recent randomized trials in patients with early-stage periodontal disease or established cardiovascular disease (Okada et al. [25], Molina et al. [39]) did not demonstrate significant FMD benefits, contributing to the substantial heterogeneity (I^2^ = 77%).

### 3.8. Heterogeneity and Effect Modifiers

Moderate statistical heterogeneity was observed in the pooled analyses (I^2^ = 77% for the primary FMD outcome). This heterogeneity appears to be driven primarily by differences in periodontal disease severity, treatment intensity (intensive vs. conventional non-surgical therapy), follow-up duration (short-term ≤3 months vs. longer-term), baseline cardiovascular risk profile of the participants, and, to a lesser extent, the specific methods used to assess endothelial function.

Beyond clinical and methodological sources of variability, underlying genetic heterogeneity across study populations may represent an additional contributor to the observed heterogeneity in FMD responses. Polygenic variation in genes encoding key inflammatory mediators—including interleukin-6 (IL-6) and C-reactive protein (CRP)—has been associated with inter-individual differences in endothelial function and vascular reactivity [38]. Such genetic variability was not assessed or adjusted for in the included studies, and may partly explain the heterogeneity in FMD outcomes that could not be fully accounted for by clinical or methodological factors alone.

Studies enrolling patients with more advanced periodontal disease (e.g., Stage III–IV) or higher baseline cardiovascular risk (such as hypertension or established cardiovascular disease) tended to report larger absolute improvements in flow-mediated dilation (FMD) and greater reductions in inflammatory markers following periodontal therapy. In contrast, trials involving patients with early-stage periodontal disease (e.g., Okada et al., 2021 [25]) or those with established cardiovascular disease under optimized management (e.g., Molina et al., 2025 [39]) showed smaller or non-significant effects.

Cochran’s Q test confirmed the presence of heterogeneity (*p* < 0.05). Despite this variability, the overall direction of effect generally favored improved endothelial function, although some studies reported neutral findings. Sensitivity analyses (leave-one-out and restriction to randomized controlled trials) did not materially alter the overall interpretation, supporting the robustness of the pooled estimate despite moderate heterogeneity.

Formal subgroup analyses according to periodontitis severity or treatment protocol were not performed due to the limited number of studies and inconsistent reporting of standardized staging, as many studies predated the 2017 AAP/EFP classification. Nevertheless, the observed pattern suggests that greater vascular and inflammatory benefits may be associated with higher baseline disease severity and systemic inflammatory burden.

### 3.9. Publication Bias

Visual inspection of the funnel plot for the primary FMD outcome did not suggest substantial asymmetry (Supplementary Figure S1). Egger’s regression test (*p* = 0.18) and Begg’s rank correlation test did not indicate statistically significant small-study effects.

However, these findings should be interpreted with caution. Methodological guidance indicates that tests for funnel plot asymmetry have limited statistical power when fewer than ten studies are included in a meta-analysis. Given the small number of studies contributing to the quantitative synthesis, the absence of apparent asymmetry does not exclude the possibility of publication or reporting bias.

## 4. Discussion

### 4.1. Principal Findings

This systematic review and meta-analysis provides a comprehensive synthesis of the relationship between periodontal disease, periodontal therapy, and endothelial function. Three principal findings emerge. First, periodontitis is associated with impaired endothelial function compared with periodontally healthy individuals, supporting the concept that chronic oral inflammation contributes to systemic vascular dysfunction. Second, periodontal therapy is associated with statistically significant improvements in endothelial function, as assessed by flow-mediated dilation (FMD), although the magnitude of benefit varies across populations and study designs. Third, periodontal treatment is accompanied by reductions in systemic inflammatory biomarkers, particularly C-reactive protein (CRP) and interleukin-6 (IL-6), suggesting that vascular improvements may be mediated, at least in part, through attenuation of systemic inflammation.

The pooled mean difference in FMD after periodontal therapy of 3.3 percentage points (95% CI 1.7–4.9) indicates a biologically relevant enhancement of endothelial function. Importantly, this estimate reflects absolute percentage-point changes rather than relative proportional differences. Larger absolute improvements were typically observed in earlier interventional studies that enrolled patients with markedly impaired baseline endothelial function, consistent with the concept that individuals with greater vascular dysfunction may derive greater benefit from periodontal therapy. In contrast, more recent randomized trials in patients with early-stage periodontal disease or in those with established but well-managed cardiovascular disease (Okada et al. [25]; Molina et al. [39]) demonstrated smaller or non-significant changes in FMD despite clear improvements in periodontal parameters. These findings suggest that baseline vascular status, degree of endothelial impairment, and overall cardiovascular risk profile may substantially modulate the magnitude of endothelial response to periodontal treatment.

Although endothelial function is a well-established surrogate marker of vascular health, the present findings are based on functional vascular endpoints rather than hard clinical outcomes. Previous prospective cohort studies have suggested that approximately a 1% absolute increase in FMD is associated with an estimated 10–13% lower risk of future cardiovascular events [1,2,3]. However, extrapolation of these associations to the present findings should be made cautiously. The included studies were not designed or powered to evaluate long-term cardiovascular outcomes, and the observed changes in endothelial function cannot be directly translated into quantitative estimates of cardiovascular risk reduction.

Taken together, the available evidence supports the hypothesis that periodontal therapy may represent a modifiable intervention capable of improving early vascular dysfunction and reducing systemic inflammatory burden. While the clinical implications for cardiovascular event prevention remain to be fully established, the observed improvements in endothelial function and inflammatory biomarkers support the biological plausibility that comprehensive periodontal care could contribute to cardiovascular risk modification as part of an integrated preventive strategy.

### 4.2. Biological Mechanisms Linking Periodontitis and Endothelial Dysfunction

The association between periodontitis and endothelial dysfunction is biologically plausible and supported by several converging mechanistic pathways. Periodontitis is a chronic inflammatory condition characterized by a dysbiotic subgingival biofilm and sustained host immune activation, leading to recurrent bacteremia, endotoxemia, and systemic dissemination of pro-inflammatory mediators [4,5,6]. Circulating cytokines, including interleukin-6 (IL-6) and tumor necrosis factor-α, together with acute-phase proteins such as C-reactive protein (CRP), promote endothelial activation, oxidative stress, reduced nitric oxide bioavailability, and impaired vasodilatory function, thereby favoring a pro-atherogenic vascular phenotype [5,7].

Additional mechanisms have been proposed, including direct interaction between periodontal pathogens and the vascular endothelium, molecular mimicry and immune cross-reactivity, amplification of oxidative stress, and prothrombotic alterations in platelet function and coagulation [6,8]. A comprehensive review of the molecular pathways linking periodontitis to vascular endothelial dysfunction has further highlighted the role of periodontal pathogens—particularly *Porphyromonas gingivalis*—in directly activating endothelial inflammatory signaling cascades [42]. Importantly, interventional evidence indicates that periodontal therapy is accompanied by concurrent reductions in systemic inflammatory biomarkers and improvements in endothelial function in several studies [30,31,32,35,38,39], supporting a mechanistic link between local infection control and systemic vascular responses. These observations are consistent with the broader concept that systemic inflammation and endothelial dysfunction represent shared pathophysiological pathways across multiple chronic inflammatory conditions.

Notably, the magnitude of vascular response to periodontal therapy appears to be influenced by baseline disease severity and systemic inflammatory burden. Studies enrolling patients with more advanced periodontal disease and greater baseline endothelial impairment tend to demonstrate larger improvements in endothelial function following treatment, whereas trials in patients with early-stage periodontal disease or established but well-controlled cardiovascular conditions have reported more modest or neutral effects on flow-mediated dilation [24,25,32,33,36,39]. This pattern is consistent with the concept that the reversibility of endothelial dysfunction depends on the baseline degree of vascular impairment and inflammatory activation, with greater potential for improvement in those with more severe dysfunction at baseline.

Taken together, these findings support the concept that periodontal inflammation may contribute to vascular dysfunction primarily through systemic inflammatory pathways, with additional contributions from direct microbial and prothrombotic mechanisms. While a direct causal relationship between periodontitis and adverse cardiovascular outcomes cannot be definitively established based on the available data, the observed improvements in endothelial function and inflammatory markers following periodontal therapy are consistent with a biologically plausible link between periodontal disease and vascular health.

### 4.3. Interpretation of Interventional Findings

The interventional evidence provides important insights into the relationship between periodontal therapy and endothelial function. The randomized trial by Tonetti et al. demonstrated a biphasic vascular response following intensive periodontal therapy, with transient early endothelial impairment followed by sustained improvement over several months [24]. This pattern likely reflects an acute inflammatory response to mechanical intervention, followed by a longer-term reduction in systemic inflammatory burden. Consistent improvements in endothelial function were also reported in several independent cohorts treated with non-surgical periodontal therapy [32,33,34,36], supporting the reproducibility of vascular benefits across different clinical settings.

The observation that endothelial recovery is frequently accompanied by reductions in CRP, IL-6, and fibrinogen supports an inflammation-mediated mechanism of vascular improvement [33,37,38]. However, the certainty of evidence for IL-6 reduction should be interpreted cautiously. According to the GRADE framework, the available evidence for IL-6 was rated as very low certainty due to study limitations, heterogeneity in biomarker reporting, and imprecision related to the small number of contributing studies, and should therefore be considered hypothesis-generating rather than definitive.

Importantly, the improvement in endothelial function observed following periodontal therapy may reflect not only local control of oral inflammation but also systemic modulation of inflammatory signaling pathways. Periodontal treatment has been shown to reduce circulating levels of inflammatory mediators and oxidative stress markers, which in turn may restore endothelial nitric oxide synthase activity and improve vascular reactivity.

This supports the concept that periodontal therapy may exert beneficial vascular effects through a combination of anti-inflammatory and endothelial-protective mechanisms, positioning periodontal disease as a modifiable contributor to systemic vascular dysfunction within the broader framework of chronic inflammatory conditions.

Not all interventional studies demonstrated a significant improvement in endothelial function. More recent randomized trials conducted in patients with early-stage periodontal disease or in individuals with established but well-controlled cardiovascular disease showed smaller or non-significant changes in FMD despite clear improvements in periodontal parameters [25,39]. These findings suggest that baseline endothelial function, periodontal disease severity, and cardiovascular risk profile may influence the magnitude of vascular response to periodontal therapy, with a smaller dynamic range for improvement in patients who have relatively preserved endothelial function or intensive background cardiovascular management.

The magnitude of the pooled FMD improvement observed in the present analysis should therefore be interpreted with caution. Inclusion of four additional recent studies resulted in a more conservative pooled estimate of 3.3 percentage points. This value primarily supports the direction and biological plausibility of the association between periodontal therapy and endothelial function improvement, rather than serving as a precise quantitative prediction of the expected clinical effect size. Larger improvements in earlier mechanistic trials likely reflect both genuine vascular recovery and regression toward the mean in patients with severe baseline endothelial dysfunction, while more recent trials in milder or higher-risk populations showed attenuated effects.

This pooled effect size also appears greater than the improvements typically reported in contemporary pharmacological cardiovascular intervention studies. This discrepancy likely reflects methodological differences between early mechanistic periodontal trials and more recent rigorously controlled clinical studies. Several of the earlier investigations enrolled small cohorts with marked baseline endothelial impairment and relied heavily on within-group changes rather than strictly controlled between-group comparisons. In such contexts, larger absolute improvements in FMD are biologically plausible following a reduction in inflammatory burden but should not be interpreted as directly comparable to effect sizes observed in large-scale cardiovascular outcome trials.

### 4.4. Comparison with Previous Meta-Analyses

The present findings are broadly consistent with previous quantitative syntheses investigating the relationship between periodontal disease, periodontal therapy, and vascular function. Earlier meta-analyses have reported that periodontal therapy is associated with improvements in endothelial function and reductions in systemic inflammatory markers, although substantial heterogeneity was noted due to variability in study design, populations, and treatment protocols. For example, Teeuw et al. demonstrated significant improvements in vascular surrogate outcomes and inflammatory biomarkers following periodontal therapy, while Orlandi et al. confirmed an association between periodontal treatment and improved endothelial function, highlighting systemic inflammation as a potential mediating mechanism [43,44]. A large meta-analysis of 26 randomized controlled trials by Luthra et al. (2023) [45] reported a pooled reduction in C-reactive protein of 0.69 mg/L following periodontal treatment, providing high-quality evidence corroborating the systemic anti-inflammatory effect observed in our analysis. More recently, Polizzi et al. (2025) [46] demonstrated that non-surgical periodontal treatment also improves arterial stiffness-related outcomes including carotid intima–media thickness and pulse wave velocity, extending the vascular benefits of periodontal therapy beyond endothelial function. Our findings are also consistent with those of Lyu et al. (2024) [5], who reported that periodontal therapy is associated with improvements in endothelial function. However, similar to our analysis, their results were characterized by substantial heterogeneity and appeared to be influenced by study design, baseline periodontal severity, and population characteristics. Notably, both analyses suggest that the magnitude of endothelial improvement may be attenuated in more recent studies or in populations with less severe periodontal disease or established cardiovascular conditions, in which baseline endothelial dysfunction is less pronounced.

More recent studies have further suggested that periodontal therapy may influence additional surrogate cardiovascular outcomes, including carotid intima–media thickness and arterial stiffness, indicating a broader vascular impact beyond endothelial function alone [5]. These observations support the concept that periodontal disease can affect multiple aspects of vascular health, ranging from functional endothelial responses to early structural arterial changes.

### 4.5. Clinical Implications

The findings of this meta-analysis have several clinically relevant implications. Endothelial dysfunction represents an early and potentially reversible stage of atherosclerosis; therefore, the observation that periodontal therapy is associated with improvements in endothelial function suggests that oral health interventions may contribute to cardiovascular prevention strategies [1,2,3]. Supporting the clinical relevance of these findings, a large meta-analysis of 39 prospective cohort studies including over 4 million individuals demonstrated that periodontal disease is independently associated with increased risk of major adverse cardiovascular events, stroke, and all-cause mortality [47]. Notably, this association between periodontal disease and cardiovascular risk has been shown to be independent of sex, further supporting the universality of the oral-systemic link across diverse populations [48]. Given the high global prevalence of periodontitis and its frequent underdiagnosis, integration of periodontal assessment into cardiovascular risk evaluation may represent a potentially cost-effective preventive approach.

Patients with established cardiovascular risk factors, including hypertension, appear to derive comparable or potentially greater vascular benefits from periodontal therapy [31,38]. However, the magnitude of response varies across populations, and more modest or neutral effects have been observed in individuals with less advanced periodontal disease or lower baseline inflammatory burden [24,25,32,33,36,39]. These findings suggest that baseline endothelial function, periodontal disease severity, and cardiovascular risk profile may influence the degree of vascular improvement following periodontal therapy.

Periodontal therapy should be considered complementary to, rather than a substitute for, established cardiovascular prevention strategies, including pharmacological risk factor control and lifestyle modification. Nevertheless, interventions that reduce systemic inflammation and improve endothelial function—such as periodontal treatment—may contribute to broader cardiovascular risk reduction strategies [33,37,38].

From a practical clinical perspective, these findings support closer collaboration between cardiology and dental care providers. For cardiologists, the presence of moderate-to-severe periodontitis may serve as a marker of increased systemic inflammatory burden and endothelial dysfunction [5,7]. Although periodontal disease is not currently incorporated into formal cardiovascular risk prediction models, awareness of periodontal status may contribute to a more comprehensive assessment of vascular risk, particularly in patients with multiple inflammatory comorbidities.

For dental practitioners, these results provide an opportunity to emphasize the potential systemic implications of periodontal disease. In patients with established cardiovascular disease or elevated cardiovascular risk, periodontal therapy may offer benefits that extend beyond oral health, including potential improvements in endothelial function and systemic inflammatory status [24,25,32,33,36,39].

Overall, interdisciplinary collaboration between cardiologists and dental professionals may represent an important component of future preventive strategies aimed at reducing systemic inflammatory burden and improving vascular health.

### 4.6. Sources of Heterogeneity

The moderate heterogeneity observed across pooled analyses likely reflects several clinical and methodological differences among the included studies. First, treatment intensity varied substantially, ranging from initial non-surgical periodontal therapy to intensive multi-session treatment protocols. More intensive interventions, such as those applied in the randomized trial by Tonetti et al. [24], were associated with larger and more sustained improvements in endothelial function, suggesting that the magnitude of vascular recovery may depend on the degree of periodontal inflammation reduction.

Second, follow-up duration differed considerably across studies. Some investigations assessed endothelial responses within days or weeks after therapy, while others evaluated vascular function after several months. Because endothelial recovery may evolve over time following a reduction in systemic inflammatory burden, differences in follow-up timing may contribute to variability in reported FMD improvements.

Third, the cardiovascular risk profile and baseline inflammatory status of the included populations varied substantially. Several studies enrolled otherwise healthy individuals with periodontitis, whereas others included patients with hypertension or established cardiovascular disease. Studies including patients with early-stage periodontal disease or with established but well-controlled cardiovascular disease tended to report smaller or non-significant changes in endothelial function, whereas those enrolling individuals with more severe periodontal destruction and higher inflammatory burden demonstrated larger absolute improvements in FMD [24,25,32,33,36,39]. These findings suggest that baseline vascular dysfunction and inflammatory status may influence the magnitude of treatment response.

Finally, methodological differences in the assessment of endothelial function may also contribute to heterogeneity. Although most studies used brachial artery FMD, one study employed forearm blood flow plethysmography to assess endothelium-dependent vasodilation [31], and minor variations in measurement protocols, operator expertise, and ultrasound techniques may influence the magnitude of reported endothelial responses. Taken together, these factors likely explain the moderate heterogeneity observed in the pooled analyses and highlight the importance of interpreting pooled estimates within the clinical context of the included studies.

Subgroup analyses according to the severity of periodontitis or type of periodontal treatment could not be reliably performed. Most included studies were conducted before the introduction of the 2017 AAP/EFP staging and grading classification and reported periodontal diagnoses using heterogeneous historical definitions. In addition, treatment protocols were not consistently categorized into comparable therapeutic strategies. As a result, subgroup stratification would have introduced substantial misclassification and statistical instability, limiting the interpretability of such analyses.

An additional, underappreciated source of heterogeneity may relate to genetic variability across study populations. Polygenic differences in inflammatory pathways—particularly variants in IL-6 and CRP—have been shown to influence baseline endothelial function and the magnitude of vascular responses independently of clinical disease burden [49]. Although this dimension could not be assessed within the present analysis, future studies should consider incorporating genetic profiling to better characterize inter-individual variability in vascular responses to periodontal therapy.

### 4.7. Strengths of the Study

This study has several important strengths. First, it was conducted using a comprehensive systematic search strategy and adhered to contemporary reporting standards (PRISMA 2020), ensuring methodological transparency and reproducibility. Second, the analysis integrates both observational and interventional evidence within a single framework, allowing a more complete evaluation of the relationship between periodontal disease, periodontal therapy, and vascular function.

Methodological rigor was further supported by the use of validated tools for risk-of-bias assessment and by evaluation of the certainty of evidence using the GRADE framework. Finally, sensitivity analyses, including leave-one-out procedures and restriction to randomized controlled trials, confirmed the robustness and consistency of the main findings, strengthening confidence in the overall conclusions.

### 4.8. Limitations

Several limitations of this systematic review and meta-analysis should be acknowledged. First, despite an updated search window extending to March 2026, the number of studies providing extractable quantitative data on endothelial function (primarily FMD) remains limited. Although more recent investigations have been published, only a subset reported FMD outcomes in a format suitable for quantitative pooling. In addition, much of the contemporary literature has focused on alternative vascular surrogate markers—such as arterial stiffness, carotid intima–media thickness, and circulating inflammatory biomarkers—rather than direct assessment of endothelial nitric oxide-mediated vasodilation, and therefore did not meet the predefined inclusion criteria. Given the limited number of FMD studies and substantial heterogeneity (I^2^ = 77%), the pooled improvement of 3.3 percentage points should be interpreted as an exploratory signal of benefit rather than a robust quantitative estimate.

Second, the total number of included studies and individual sample sizes were relatively modest, which may limit statistical precision. Clinical heterogeneity in periodontal diagnostic criteria and treatment protocols likely influenced pooled estimates. Most studies were conducted before the introduction of the 2017 AAP/EFP staging and grading classification and reported periodontal disease using heterogeneous historical definitions, further contributing to variability. In addition, most investigations evaluated surrogate vascular endpoints rather than hard cardiovascular outcomes, precluding direct conclusions regarding long-term clinical benefit.

Third, the quantitative synthesis combined randomized and non-randomized interventional studies. While this approach increases statistical power in a field with limited randomized evidence, it introduces methodological heterogeneity and potential confounding. Nevertheless, sensitivity analyses restricted to randomized controlled trials demonstrated a consistent direction of effect, suggesting that the main findings were not driven solely by non-randomized designs. Residual confounding cannot be excluded in observational data despite adjustment for conventional cardiovascular risk factors. From a methodological perspective, the overall certainty of evidence for FMD was graded as moderate primarily because of serious risk of bias. Several influential interventional studies were non-randomized and evaluated within-group pre–post changes (ROBINS-I: moderate risk of bias), and even among randomized trials, open-label designs and performance bias (e.g., Okada) introduced additional concerns. These limitations reduce confidence in the exact magnitude of the pooled effect, even though the overall direction of benefit appears consistent across study designs.

Fourth, follow-up durations were generally short to intermediate, limiting assessment of the durability of vascular effects over longer time horizons. In addition, formal power calculations were not consistently reported, which may affect the precision of effect estimates. Although formal tests and visual inspection did not indicate clear publication bias, these methods have limited power when based on a small number of studies; therefore, publication or reporting bias cannot be definitively excluded. The restriction to English-language studies may also have introduced language bias.

Given the limited number of studies included in the quantitative synthesis, the statistical power of publication bias assessment remains low, and these findings should be interpreted cautiously. More broadly, the inclusion of only six studies in the quantitative meta-analysis constitutes a substantive limitation with respect to both statistical power—reducing the precision of pooled estimates and the ability to detect true effects of moderate magnitude—and the reliability of formal publication bias detection. With so few studies, Egger’s test and visual funnel plot inspection are known to have low sensitivity, meaning that the absence of detected asymmetry should not be interpreted as evidence against the presence of publication or small-study bias.

Finally, grey literature and non-peer-reviewed sources were not systematically included. Although such sources may increase the number of identified records, they rarely provide sufficient methodological detail or quantitative data for reliable synthesis. Overall, these limitations indicate that the pooled estimates should be interpreted primarily as evidence of directionality and biological plausibility rather than precise quantitative measures of the vascular benefit of periodontal therapy.

### 4.9. Future Research Directions

Future research should prioritize large, well-designed randomized controlled trials evaluating the impact of periodontal therapy on hard cardiovascular outcomes, including major adverse cardiovascular events (MACE) such as myocardial infarction, stroke, and cardiovascular mortality. Longer follow-up periods are essential to determine whether improvements observed in surrogate vascular markers, particularly flow-mediated dilation, translate into sustained reductions in clinical cardiovascular risk.

Standardization of periodontal diagnostic criteria and treatment protocols represents another key priority, as heterogeneity in disease definitions and therapeutic approaches currently limits comparability across studies. In addition, future investigations should incorporate comprehensive biomarker profiling to better characterize the mechanistic pathways linking periodontal inflammation to endothelial dysfunction and systemic vascular effects.

Particular attention should be given to high-risk populations, including patients with established cardiovascular disease or multiple cardiometabolic comorbidities, in whom periodontal therapy may have the greatest potential clinical impact. Evaluating periodontal treatment as an adjunct to conventional cardiovascular prevention strategies in these groups may provide important insights into its role in integrated risk management.

## 5. Conclusions

In conclusion, this systematic review and meta-analysis indicates that periodontitis is associated with impaired endothelial function and increased systemic inflammation, whereas periodontal therapy is associated with measurable improvements in vascular function and inflammatory biomarkers in a range of clinical settings. These findings support the concept that periodontal disease represents a potentially modifiable contributor to systemic vascular dysfunction, acting at least in part through inflammatory pathways. Given the limitations of the available evidence and the reliance on surrogate endpoints, the pooled estimates should be interpreted primarily as evidence of directionality and biological plausibility rather than precise quantitative predictions of clinical benefit.

## Figures and Tables

**Table 1:** Characteristics of the included studies.

First Author, Year	Country	Study Design	Population (N, Mean Age, % Male)	Periodontitis Definition/Severity	Comparator Group	Periodontal Intervention	Endothelial Function Assessment	Follow-Up Duration
Amar, 2003 [30]	USA	Case–control	79 adults; middle-aged; balanced sex distribution	Chronic periodontitis defined by clinical attachment loss and probing depth	Periodontally healthy controls	Not applicable (baseline association)	Brachial artery FMD	Cross-sectional (single assessment)
Mercanoglu, 2004 [32]	Turkey	Interventional (non-randomized)	40 adults with chronic periodontitis	Chronic periodontitis	Within-patient comparison	Scaling and root planing	Brachial artery FMD	Short-term post-treatment
Ide, 2004 [35]	UK	Interventional (non-randomized)	30 adults with chronic periodontitis	Chronic periodontitis	Within-patient comparison	Scaling and root planing	Systemic inflammatory markers (CRP, TNF-α, IL-6; no direct endothelial function measurement)	Short-term
Seinost, 2005 [33]	Austria	Interventional (non-randomized)	30 patients with severe generalized periodontitis	Severe generalized periodontitis	Baseline comparison with controls	Non-surgical periodontal therapy	Brachial artery FMD	3 months after therapy
D’Aiuto, 2004 [37]	UK	Randomized clinical trial	94 patients with severe periodontitis	Severe periodontitis	Control group (less intensive therapy)	Intensive vs. control periodontal therapy	Systemic inflammatory markers (CRP, IL-6; no direct endothelial function measurement)	6 months
Tonetti, 2007 [24]	UK	Randomized controlled trial	120 patients with severe generalized periodontitis	Severe generalized periodontitis	Control group receiving standard therapy	Intensive periodontal therapy vs. control regimen	Brachial artery FMD	24 h, 60 days, 180 days
Blum, 2007 [34]	Israel	Interventional (non-randomized)	13 patients with periodontitis; 10 healthy controls	Moderate–severe periodontitis	Healthy controls	Non-surgical periodontal therapy	Brachial artery FMD	3 months after therapy
Higashi, 2008 [31]	Japan	Cross-sectional (mechanistic)	96 participants (normotensive and hypertensive)	Periodontitis diagnosed clinically (presence vs. absence)	Subjects without periodontitis	Not applicable	Forearm blood flow plethysmography (endothelium-dependent and independent vasodilation)	Cross-sectional (single assessment)
Piconi, 2009 [36]	Italy	Interventional (non-randomized)	40 adults with chronic periodontitis	Chronic periodontitis	Within-patient comparison	Periodontal therapy	Brachial artery FMD and carotid intima–media thickness (CIMT)	1, 6, and 12 months after therapy
Vidal, 2009 [38]	Brazil	Interventional (non-randomized)	77 patients with severe periodontitis and refractory hypertension	Severe periodontitis	Within-patient comparison	Periodontal therapy	Systemic inflammatory markers (IL-6, CRP, fibrinogen; no direct endothelial function measurement)	Months after therapy
Holtfreter, 2013 [41]	Germany	Cross-sectional population-based study	1234 participants; 25–85 years; ~50% male	Periodontitis defined by clinical attachment loss and probing depth (standardized periodontal examination)	Participants without periodontitis or with lower disease severity	Not applicable	Brachial artery FMD and nitroglycerin-mediated dilation (NMD) assessed by ultrasound	Cross-sectional (single assessment)
Okada, 2021 [25]	Japan	Randomized controlled trial (open-label)	110 patients with early-stage periodontal disease	Early-stage periodontal disease	Control group (standard oral hygiene)	Advanced periodontal self-care intervention	Brachial artery FMD	3 months
Velosa-Porras, 2021 [40]	Colombia	Case–control observational study	202 adults (101 cases, 101 controls)	Stage III–IV periodontitis (cases) vs. healthy/gingivitis (controls)	Periodontally healthy or gingivitis controls	Not applicable	Brachial artery flow-mediated dilation (FMD) assessed by ultrasound	Cross-sectional (single assessment)
Molina, 2025 [39]	Spain	Randomized pilot clinical trial	35 patients with periodontitis and established cardiovascular disease	Moderate–severe periodontitis	Control group (standard care)	Non-surgical periodontal therapy	Brachial artery FMD and serum biomarkers	3 months

**Table 2:** Study-level endothelial function (FMD) data across included studies (qualitative synthesis).

First Author, Year	Study Design/Population	Periodontal Intervention vs. Comparator	Baseline FMD (%)	Post-Treatment/Comparator FMD (%)	Absolute Change (ΔFMD, %)	*p*-Value	Notes
Amar, 2003 [30]	Case–control; periodontitis vs. healthy controls	No active therapy	7.8 ± 4.6 vs. 11.7 ± 5.3	Not applicable	−3.9	0.005	Lower FMD in periodontitis
Mercanoglu, 2004 [32]	Interventional; chronic periodontitis	Non-surgical therapy (within-patient)	8.4 ± 4.0	17.7 ± 5.7	+9.3	<0.001	Marked improvement
Seinost, 2005 [33]	Interventional; severe periodontitis	Non-surgical therapy	6.1 ± 4.4	9.8 ± 5.7	+3.7	0.003	Improved FMD and reduced CRP
Tonetti, 2007 [24]	RCT; severe generalized periodontitis	Intensive vs. standard therapy	Similar in both groups	+2.0 (180 days, between-group)	+2.0	<0.001 (180 days)	Biphasic response
Blum, 2007 [34]	Interventional; mixed CV risk	Non-surgical therapy	4.12 ± 3.96	11.12 ± 7.22	+7.0	0.007	Significant improvement
Piconi, 2009 [36]	Interventional; chronic periodontitis	Periodontal therapy	Impaired at baseline	Improved after therapy	Improved	<0.01	Also reduced CIMT
Okada, 2021 [25]	Open-label RCT; early-stage disease	Self-care vs. standard	~5.5–5.8	No significant change	−0.2 (between-group)	0.708	No FMD benefit
Molina, 2025 [39]	Randomized pilot RCT; periodontitis + CVD	Therapy vs. standard care	13.35/15.06	9.72/10.14	−3.63/−4.92	>0.05 (between-group)	No significant FMD benefit
Holtfreter, 2013 [41]	Cross-sectional population-based	No intervention	Lower with increasing disease severity	Not applicable	Lower FMD with increasing severity	<0.05 (after adjustment)	Independent association
Velosa-Porras, 2021 [40]	Case–control; stage III–IV periodontitis	No intervention	Lower in periodontitis group	Not applicable	Lower FMD in periodontitis	>0.05 (after adjustment)	No significant association after adjustment

Note: ΔFMD represents the absolute change in flow-mediated dilation. For interventional studies, it was calculated as the difference between post-treatment and baseline mean values. For randomized controlled trials, between-group differences are reported where applicable.

**Table 3:** Study-level changes in systemic inflammatory biomarkers following periodontal therapy.

First Author, Year	Study Design/Population	Periodontal Intervention vs. Comparator	Biomarker	Baseline Value	Post-Treatment Value	Absolute Change (Δ)	*p*-Value
Amar, 2003 [30]	Case–control; periodontitis vs. healthy controls	No active therapy	CRP	2.3 ± 2.3 vs. 1.0 ± 1.0 mg/L	Not applicable	+1.3 mg/L	0.03
Ide, 2004 [35]	Interventional; chronic periodontitis	Scaling and root planing (within-patient)	IL-6	Not reported	Return toward baseline	Transient increase followed by normalization	Not applicable
Ide, 2004 [35]	Interventional; chronic periodontitis	Scaling and root planing (within-patient)	CRP	Not reported	Return toward baseline	Transient increase followed by normalization	Not applicable
D’Aiuto, 2004 [37]	RCT; severe periodontitis	Intensive vs. control therapy	IL-6	Not reported	Not reported	−0.2 ng/L (95% CI 0.1–0.4)	0.001
D’Aiuto, 2004 [37]	RCT; severe periodontitis	Intensive vs. control therapy	CRP	Not reported	Not reported	−0.5 mg/L (95% CI 0.4–0.7)	<0.0001
Seinost, 2005 [33]	Interventional; severe periodontitis	Non-surgical therapy	CRP	1.1 ± 1.9 mg/L	0.8 ± 0.8 mg/L	−0.3 mg/L	0.026
Vidal, 2009 [38]	Interventional; severe periodontitis + refractory hypertension	Periodontal therapy	IL-6	Elevated	Reduced	Reduced	<0.05
Vidal, 2009 [38]	Interventional; severe periodontitis + refractory hypertension	Periodontal therapy	CRP	Elevated	Reduced	Reduced	<0.05
Vidal, 2009 [38]	Interventional; severe periodontitis + refractory hypertension	Periodontal therapy	Fibrinogen	Elevated	Reduced	Reduced	<0.05
Molina, 2025 [39]	Randomized pilot RCT; periodontitis + CVD	Therapy vs. standard care	CRP	Not reported	Not reported	Numerical reduction	>0.05 (between-group)
Molina, 2025 [39]	Randomized pilot RCT; periodontitis + CVD	Therapy vs. standard care	IL-6	Not reported	Not reported	Numerical reduction	>0.05 (between-group)

Note: Δ represents the absolute change in biomarker levels following periodontal therapy. For interventional studies, changes refer to differences between post-treatment and baseline values or between-group differences where applicable. “Not reported” indicates that numerical values were not provided in the original study, while “Not applicable” denotes outcomes not suitable for quantitative comparison (e.g., transient inflammatory responses).

## Data Availability

No new data were created or analyzed in this study. Data sharing is not applicable to this article.

## Supplementary Materials

The following supporting information can be downloaded at: https://www.mdpi.com/article/10.3390/biomedicines14051106/s1, Supplementary Table S1: PRISMA checklist; Supplementary Table S2: Detailed search strategy for each database; Supplementary Table S3: Risk of bias assessment of included studies; Supplementary Table S4: GRADE Summary of Findings: Effect of Periodontal Therapy on Endothelial Function and Systemic Inflammatory Markers; Supplementary Figure S1: Funnel plot assessing potential publication bias for the primary outcome.

## Abbreviations

The following abbreviations are used in this manuscript:

ACh	Acetylcholine
CI	Confidence interval
CRP	C-reactive protein
FMD	Flow-mediated dilation
GRADE	Grading of Recommendations Assessment, Development and Evaluation
IL-6	Interleukin-6
NOS	Newcastle–Ottawa Scale
PRISMA	Preferred Reporting Items for Systematic Reviews and Meta-Analyses
PROSPERO	International Prospective Register of Systematic Reviews
RCT	Randomized controlled trial
ROBINS-I	Risk Of Bias In Non-randomized Studies of Interventions
RoB 2	Cochrane Risk of Bias 2 tool
SNP	Sodium nitroprusside
