# Supplementary material for: Periodontitis, Endothelial Dysfunction, and Systemic Inflammation: A Systematic Review and Meta-Analysis of Flow-Mediated Dilation
Source: Biomedicines. 2026 May 14;14(5):1106. doi: 10.3390/biomedicines14051106 (PMC13204477; doi:10.3390/biomedicines14051106)
Supplement: Supplementary file 1 [file biomedicines-14-01106-s001.zip › biomedicines-4279028-supplementary.pdf]

Supplementary Table S1. PRISMA checklist.

Section and Topic	Item #	Checklist item	Location where item is reported
**TITLE**			
Title	1	Identify the report as a systematic review.	Title page — “Systematic Review and Meta-Analysis” stated above the manuscript title
**ABSTRACT**			
Abstract	2	See the PRISMA 2020 for Abstracts checklist.	Structured Abstract (entire Abstract section)
**INTRODUCTION**			
Rationale	3	Describe the rationale for the review in the context of existing knowledge.	Introduction, paragraphs 1–4 (periodontitis, systemic inflammation, endothelial dysfunction, cardiovascular implications)
Objectives	4	Provide an explicit statement of the objective(s) or question(s) the review addresses.	Introduction, final paragraph (explicit aim of systematic review and meta-analysis)
**METHODS**			
Eligibility criteria	5	Specify the inclusion and exclusion criteria for the review and how studies were grouped for the syntheses.	Materials and Methods → Section 2.3 (Eligibility Criteria)
Information sources	6	Specify all databases, registers, websites, organisations, reference lists and other sources searched or consulted to identify studies. Specify the date when each source was last searched or consulted.	Materials and Methods → Section 2.4 (Databases and last search date March 2026)
Search strategy	7	Present the full search strategies for all databases, registers and websites, including any filters and limits used.	Supplementary Table S2 (Detailed Search Strategy)
Selection process	8	Specify the methods used to decide whether a study met the inclusion criteria of the review, including how many reviewers screened each record and each report retrieved, whether they worked independently, and if applicable, details of automation tools used in the process.	Materials and Methods → Section 2.5 (two independent reviewers)
Data collection process	9	Specify the methods used to collect data from reports, including how many reviewers collected data from each report, whether they worked independently, any processes for obtaining or confirming data from study investigators, and if applicable, details of automation tools used in the process.	Materials and Methods → Section 2.6 (two reviewers independently)

Section and Topic	Item #	Checklist item	Location where item is reported
Data items	10a	List and define all outcomes for which data were sought. Specify whether all results that were compatible with each outcome domain in each study were sought (e.g. for all measures, time points, analyses), and if not, the methods used to decide which results to collect.	Materials and Methods → Section 2.2 (PICO; primary and secondary outcomes)
	10b	List and define all other variables for which data were sought (e.g. participant and intervention characteristics, funding sources). Describe any assumptions made about any missing or unclear information.	Materials and Methods → Section 2.6 (study characteristics, biomarkers, endothelial measures)
Study risk of bias assessment	11	Specify the methods used to assess risk of bias in the included studies, including details of the tool(s) used, how many reviewers assessed each study and whether they worked independently, and if applicable, details of automation tools used in the process.	Materials and Methods → Section 2.7; Supplementary Table S3
Effect measures	12	Specify for each outcome the effect measure(s) (e.g. risk ratio, mean difference) used in the synthesis or presentation of results.	Materials and Methods → Section 2.8 (mean differences with 95% CI)
Synthesis methods	13a	Describe the processes used to decide which studies were eligible for each synthesis (e.g. tabulating the study intervention characteristics and comparing against the planned groups for each synthesis (item #5)).	Materials and Methods → Sections 2.3 and 2.8
	13b	Describe any methods required to prepare the data for presentation or synthesis, such as handling of missing summary statistics, or data conversions.	Materials and Methods → Section 2.8
	13c	Describe any methods used to tabulate or visually display results of individual studies and syntheses.	Results → Figures and Tables
	13d	Describe any methods used to synthesize results and provide a rationale for the choice(s). If meta-analysis was performed, describe the model(s), method(s) to identify the presence and extent of statistical heterogeneity, and software package(s) used.	Materials and Methods → Section 2.8 (random-effects DerSimonian–Laird; I^2; Q test; software R and RevMan)
	13e	Describe any methods used to explore possible causes of heterogeneity among study results (e.g. subgroup analysis, meta-regression).	Materials and Methods → Section 2.8 (sensitivity analyses)
	13f	Describe any sensitivity analyses conducted to assess robustness of the synthesized results.	Materials and Methods → Section 2.8; Results → Section 3.7
Reporting bias assessment	14	Describe any methods used to assess risk of bias due to missing results in a synthesis (arising from reporting biases).	Materials and Methods → Section 2.8; Results → Section 3.9;

Section and Topic	Item #	Checklist item	Location where item is reported
			Supplementary Figure S1
Certainty assessment	15	Describe any methods used to assess certainty (or confidence) in the body of evidence for an outcome.	Materials and Methods → Section 2.9; Supplementary Table S4
**RESULTS**			
Study selection	16a	Describe the results of the search and selection process, from the number of records identified in the search to the number of studies included in the review, ideally using a flow diagram.	Results → Section 3.1; Figure 1 (PRISMA diagram)
	16b	Cite studies that might appear to meet the inclusion criteria, but which were excluded, and explain why they were excluded.	Results → Section 3.1
Study characteristics	17	Cite each included study and present its characteristics.	Results → Section 3.2; Table 1
Risk of bias in studies	18	Present assessments of risk of bias for each included study.	Results → Section 3.8; Supplementary Table S3
Results of individual studies	19	For all outcomes, present, for each study: (a) summary statistics for each group (where appropriate) and (b) an effect estimate and its precision (e.g. confidence/credible interval), ideally using structured tables or plots.	Results → Sections 3.4–3.6; Tables 2–3
Results of syntheses	20a	For each synthesis, briefly summarise the characteristics and risk of bias among contributing studies.	Results → Sections 3.4–3.7
	20b	Present results of all statistical syntheses conducted. If meta-analysis was done, present for each the summary estimate and its precision (e.g. confidence/credible interval) and measures of statistical heterogeneity. If comparing groups, describe the direction of the effect.	Results → Section 3.7; Figure 2
	20c	Present results of all investigations of possible causes of heterogeneity among study results.	Results → Section 3.8
	20d	Present results of all sensitivity analyses conducted to assess the robustness of the synthesized results.	Results → Section 3.7
Reporting biases	21	Present assessments of risk of bias due to missing results (arising from reporting biases) for each synthesis assessed.	Results → Section 3.9; Supplementary Figure S1
Certainty of evidence	22	Present assessments of certainty (or confidence) in the body of evidence for each outcome assessed.	Supplementary Table S4 (GRADE)
**DISCUSSION**			
Discussion	23a	Provide a general interpretation of the results in the context of other evidence.	Discussion → Sections 4.1–4.4
	23b	Discuss any limitations of the evidence included in the review.	Discussion → Section 4.8
	23c	Discuss any limitations of the review processes used.	Discussion → Section 4.8

Section and Topic	Item #	Checklist item	Location where item is reported
	23d	Discuss implications of the results for practice, policy, and future research.	Discussion → Sections 4.5 and 4.9
**OTHER INFORMATION**			
Registration and protocol	24a	Provide registration information for the review, including register name and registration number, or state that the review was not registered.	Materials and Methods → Section 2.1 (PROSPERO: CRD420261309247)
	24b	Indicate where the review protocol can be accessed, or state that a protocol was not prepared.	PROSPERO registration (no separate protocol published)
	24c	Describe and explain any amendments to information provided at registration or in the protocol.	No deviations from protocol reported
Support	25	Describe sources of financial or non-financial support for the review, and the role of the funders or sponsors in the review.	Funding Statement (“Victor Babeș University of Medicine and Pharmacy Timișoara”)
Competing interests	26	Declare any competing interests of review authors.	Conflicts of Interest Statement (“The authors declare no conflicts of interest.”)
Availability of data, code and other materials	27	Report which of the following are publicly available and where they can be found: template data collection forms; data extracted from included studies; data used for all analyses; analytic code; any other materials used in the review.	Data Availability Statement; Supplementary Materials

From: Page MJ, McKenzie JE, Bossuyt PM, Boutron I, Hoffmann TC, Mulrow CD, et al. The PRISMA 2020 statement: an updated guideline for reporting systematic reviews. BMJ 2021;372:n71. doi: 10.1136/bmj.n71. This work is licensed under CC BY 4.0. To view a copy of this license, visit https://creativecommons.org/licenses/by/4.0/

Supplementary Table S2. Detailed Search Strategy for Each Database.

Database	Search Strategy
PubMed/MEDLINE	("periodontitis"[MeSH Terms] OR periodontitis [Title/Abstract] OR "periodontal disease"[Title/Abstract] OR "periodontal diseases"[Title/Abstract])<br>AND<br>("endothelial function"[Title/Abstract] OR "endothelial dysfunction"[Title/Abstract] OR "flow mediated dilation"[Title/Abstract] OR FMD[Title/Abstract] OR "vascular function"[Title/Abstract])<br>AND<br>("periodontal therapy"[Title/Abstract] OR "periodontal treatment"[Title/Abstract] OR "scaling and root planing"[Title/Abstract] OR "nonsurgical periodontal therapy"[Title/Abstract])<br>AND

Database	Search Strategy
	("inflammation"[Title/Abstract] OR "C-reactive protein"[Title/Abstract] OR CRP[Title/Abstract] OR "interleukin-6"[Title/Abstract] OR IL-6[Title/Abstract])
Scopus	TITLE-ABS-KEY (periodontitis OR "periodontal disease" OR "periodontal diseases") AND TITLE-ABS-KEY ("endothelial function" OR "endothelial dysfunction" OR "flow mediated dilation" OR FMD OR "vascular function") AND TITLE-ABS-KEY ("periodontal therapy" OR "periodontal treatment" OR "scaling and root planing" OR "periodontal intervention") AND TITLE-ABS-KEY ("C-reactive protein" OR CRP OR "interleukin-6" OR IL-6 OR inflammation)
Web of Science Core Collection	TS = (periodontitis OR "periodontal disease" OR "periodontal diseases") AND TS = ("endothelial function" OR "endothelial dysfunction" OR "flow mediated dilation" OR FMD OR "vascular function") AND TS = ("periodontal therapy" OR "periodontal treatment" OR "scaling and root planing" OR "periodontal intervention") AND TS = ("C-reactive protein" OR CRP OR "interleukin-6" OR IL-6 OR inflammation)
Cochrane Library	(periodontitis OR "periodontal disease" OR "periodontal infection") AND ("endothelial dysfunction" OR "endothelial function" OR "flow mediated dilation" OR FMD OR "vascular function") AND ("periodontal therapy" OR "periodontal treatment" OR "scaling and root planing" OR "periodontal intervention")
Additional search methods	Manual screening of reference lists of eligible studies and relevant reviews. Google Scholar was used only for supplementary citation tracking and exploratory identification of potentially relevant articles, but not as a primary database for systematic searching. Duplicate records were removed using reference management software.
**Limits applied**	Human studies; observational and interventional clinical studies; English language (restriction applied for feasibility and consistency of data extraction); no restriction on publication year.
Last search date	March 2026

Supplementary Table S3. Risk of Bias Assessment of Included Studies.

Study (First Author, Year)	Study Design	Risk of Bias Tool	Selection Bias / Randomization	Performance Bias	Detection Bias	Attrition Bias	Reporting Bias	Confounding / Other Bias	Overall Risk of Bias
Amar, 2003	Case–control	Newcastle–Ottawa Scale (NOS)	Moderate	-	Moderate	Low	Low	Moderate	Moderate
Mercanoglu, 2004	Non-randomized interventional	ROBINS-I	Moderate	Moderate	Moderate	Low	Low	Moderate	Moderate

Study (First Author, Year)	Study Design	Risk of Bias Tool	Selection Bias / Randomization	Performance Bias	Detection Bias	Attrition Bias	Reporting Bias	Confounding / Other Bias	Overall Risk of Bias
Ide, 2004	Non-randomized interventional	ROBINS-I	Moderate	Moderate	Moderate	Low	Low	Moderate	Moderate
Seinost, 2005	Non-randomized interventional	ROBINS-I	Moderate	Moderate	Moderate	Low	Low	Moderate	Moderate
D'Aiuto, 2004/2006	Randomized controlled trial	RoB 2	Low	Low	Low	Low	Low	Low	Low
Tonetti, 2007	Randomized controlled trial	RoB 2	Low	Low	Low	Low	Low	Low	Low
Blum, 2007	Non-randomized interventional	ROBINS-I	Moderate	Moderate	Moderate	Low	Low	Moderate	Moderate
Higashi, 2008	Cross-sectional	Newcastle–Ottawa Scale (NOS)	Moderate	-	Moderate	Low	Low	Moderate	Moderate
Piconi, 2009	Non-randomized interventional	ROBINS-I	Moderate	Moderate	Moderate	Low	Low	Moderate	Moderate
Vidal, 2009	Non-randomized interventional	ROBINS-I	Moderate	Moderate	Moderate	Low	Low	Moderate	Moderate
Holtfreter, 2013	Cross-sectional population-based	Newcastle–Ottawa Scale (NOS)	Low	-	Low	Low	Low	Moderate	Moderate

Study (First Author, Year)	Study Design	Risk of Bias Tool	Selection Bias / Randomization	Performance Bias	Detection Bias	Attrition Bias	Reporting Bias	Confounding / Other Bias	Overall Risk of Bias
Okada, 2021	Open-label randomized controlled trial	RoB 2	Some concerns	High	Some concerns	Low	Low	Moderate	Moderate
Velosa-Porras, 2021	Case–control	Newcastle–Ottawa Scale (NOS)	Moderate	-	Moderate	Low	Low	Moderate	Moderate
Molina, 2025	Randomized pilot clinical trial	RoB 2	Low	Low	Low	Low	Low	Low	Low / Moderate

Supplementary Table S4. GRADE Summary of Findings: Effect of Periodontal Therapy on Endothelial Function and Systemic Inflammatory Markers.

Outcome 1: Flow-Mediated Dilation (FMD) – Primary Outcome

Certainty assessment							Summary of findings		
No. of studies (participants)	Study design	Risk of bias	Inconsistency	Indirectness	Imprecision	Publication bias	No. of patients / Effect size	Absolute effect (95% CI)	Certainty
6 studies (N ≈ 340)	RCTs and non-randomized interventional studies	Serious	Not serious	Not serious	Not serious	Not detected	Pooled mean difference in FMD after periodontal therapy	+3.3% (1.7 to 4.9)	MODERATE

Outcome 2: C-Reactive Protein (CRP) – Secondary Outcome

**Certainty assessment**						**Summary of findings**			
No. of studies (participants)	Study design	Risk of bias	Inconsistency	Indirectness	Imprecision	Publication bias	No. of patients / Effect size	Absolute effect (95% CI)	Certainty
5 studies (N ≈ 280)	RCTs and non-randomized interventional studies	Serious	Not serious	Not serious	Not serious	Not detected	Pooled mean difference in CRP after periodontal therapy	**−0.38 mg/L (−0.52 to −0.24 mg/L)**	**MODERATE**

Outcome 3: Interleukin-6 (IL-6) – Secondary Outcome

**Certainty assessment**						**Summary of findings**			
No. of studies (participants)	Study design	Risk of bias	Inconsistency	Indirectness	Imprecision	Publication bias	No. of patients / Effect size	Absolute effect (95% CI)	Certainty
3 studies (N ≈ 200)	RCTs and non-randomized interventional studies	Serious	Serious	Not serious	Serious	Not detected	Direction of effect favorable; quantitative pooling not performed due to heterogeneity	Favorable trend toward reduction (quantitative pooling limited by inconsistent reporting	**VERY LOW**

Certainty assessment	Summary of findings	
		and units)

Supplementary Figure S1. Funnel plot assessing potential publication bias for the primary outcome.

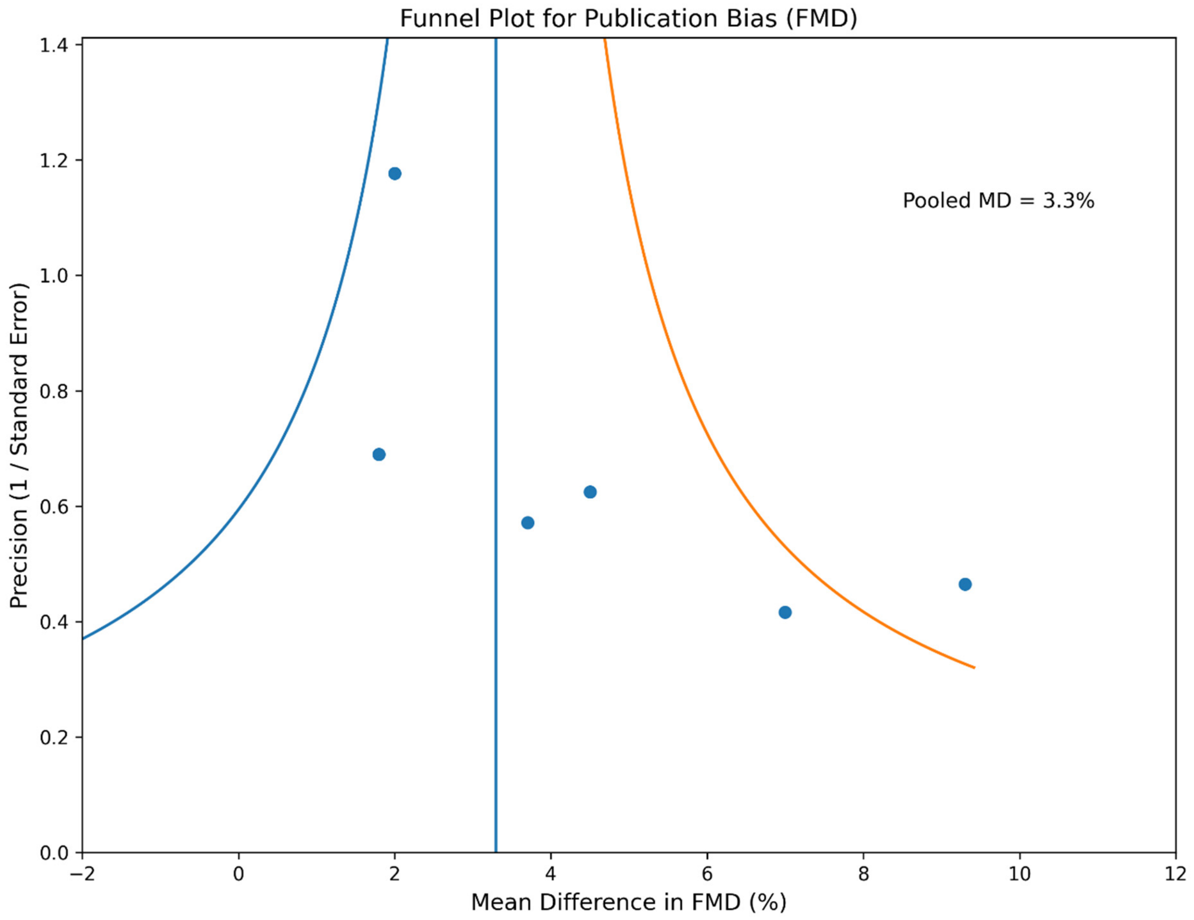

Supplementary Figure S1. Funnel plot assessing potential publication bias for studies included in the meta-analysis of flow-mediated dilation (FMD). Each point represents an individual study. The x-axis shows the effect size (mean difference in FMD), and the y-axis represents the standard error. Visual inspection suggests approximate symmetry; however, interpretation is limited due to the small number of included studies.
